# Therapeutic effect of *Periploca forrestii* on collagen-induced arthritis in rats through JAK2/Nf-κB pathway

**DOI:** 10.3389/fphar.2024.1415392

**Published:** 2024-05-22

**Authors:** Zhenyi Zhang, Yingchun Li, Jian Wu, Jihong Zhang, Ning Chen, Ning Zhang

**Affiliations:** ^1^ Department of Rheumatology and Immunology, Shengjing Hospital of China Medical University, Shenyang, China; ^2^ College of Pharmacology, Harbin University of Commerce, Harbin, China

**Keywords:** *Periploca forrestii*, arthritis, micro-CT, JAK2, NF-κB

## Abstract

**Background:**

Rheumatoid arthritis (RA) is a chronic autoimmune disease that affects the body. *Periploca forrestii* was a *miao* ethnic drug in China that was used to treat arthritis for hundreds of years. But, the therapeutic mechanism is so far unknown. Therefore, the chemical component and effect of *Periploca forrestii* on arthritis in rats were studied using HPLC-QTOF MS, micro-CT, and other experiments in this paper.

**Method:**

Male Sprague-Dawley rats were used to assess the *in vivo* activity. HPLC QTOF-MS was used to analyze the chemical profile of the *P. forrestii* (PF). Bovine type II collagen and Complete Freund’s Adjuvant were used to stimulate and construct the collagen-induced arthritis (CIA) model. Three dosages of PF (100 mg/kg, 200 mg/kg, 400 mg/kg) were used to evaluate *in vivo* activity. Methotrexate was used as the positive drug. H/E staining and micro-CT methods were used to monitor the pathological changes of CIA rats. ELISA method was used to assess the serum level of immune- and inflammation-related cytokines. Immunohistochemical experiments were used to test the gene expression in JAK and Nf-κB pathways.

**Results:**

42 compounds were identified from PF. PF administration lowered the increased spleen index compared with that of control and MTX groups, and partially restored body weight, reduced paw swelling, and arthritis score compared with the model group. Macroscopic assessment indicated inflamed paw with significant swelling in the model group, while the extent of inflammation and swelling was attenuated by both MTX and PF. H/E staining experiments demonstrated that pathological changes of synovial cells and infiltration of inflammatory cells were observed in the model group. In contrast, the MTX and PF treatment partially reversed these pathological changes. Micro-CT examination showed severe injuries and scars caused by inflammation for the model group, and in the high-dosage group (400 mg/kg) the inflammation-caused injuries and scars were dramatically ameliorated. Mechanism study showed that PF restored Nf-κB phosphorylation and JAK2 expression compared with the model group.

**Conclusion:**

*P. forrestii* possesses a potent effect on CIA rats. Nf-κB and JAK2 pathways are involved in its protective effect on CIA.

## 1 Introduction

Rheumatoid arthritis (RA) is a chronic autoimmune disease that affects the entire body. It causes swelling, inflammation, and even damage to the cartilage at the joints, which can make it difficult for people to perform daily activities. Unfortunately, the number of people suffering from RA is increasing worldwide, with up to 1% of the population being diagnosed every year (McInnes and Schett, 2017). In the past few decades, the main treatment for rheumatoid arthritis (RA) has been disease-modifying antirheumatic drugs (DMARDs). Methotrexate is the most commonly used DMARD and is considered the first choice due to its high effectiveness and affordability. However, if RA cannot be managed or if there are negative side effects to DMARDs, biological agents and targeted drugs are prescribed instead (Smolen et al., 2023).

*Periploca forrestii*, a *miao* ethnic drug in China, is a sprawling shrub that can grow as long as 10 m. It belongs to the Asclepiadaceae family and is commonly found in mountain woodlands, typically in areas with limited exposure to sunlight or among shrubbery below an altitude of 2000 m. This particular plant has been found to contain a variety of natural chemical compounds, with a total of 124 phytochemicals having been isolated and identified thus far. Among these compounds, the cardiac glycosides are particularly noteworthy due to their ability to provide a range of health benefits. These benefits include anti-inflammatory properties, as well as the ability to promote wound healing (Chen et al., 2021).

The plant can be used for medicinal purposes, particularly for dispelling wind and eliminating dampness which are closely related to RA according to the traditional medicinal theory. Clinically, it is mainly used to treat RA and traumatic injury. It has earned the nickname “The king of all lianas” for its exceptional therapeutic effect on rheumatism. However, a solid-evidenced-based investigation of *P. forrestii* on RA is still absent. Previous studies on *P. forrestii* of silico investigation or experimental investigation of a part of this plant without a clear chemical profile only preliminarily implied its potential mechanism and effects on RA (Chen et al., 2017; Wang et al., 2022). Furthermore, the effect and mechanism of the whole plant of *P. forrestii* regarding the RA is still known, which is quite important an investigation as the whole plant of *P. forrestii* (PF) is used as a traditional medicine.

## 2 Materials and methods

### 2.1 Materials

The whole plant of *P. forrestii* was purchased from Beijing TRT Group in 2019. A voucher specimen (No. PF2020041602) was deposited at the China Medical University. Antibodies were purchased from Cell Signaling Technology (USA). Micro-CT (Quantum GX PerkinElmer) was used to evaluate the pathological changes of CIA rats. ELISA kits were purchased from Abcam. Agilent 1260 HPLC-6530 QTOF MS was used to analyze the chemical profile.

### 2.2 Chemical constituents identified from *Periploca forrestii* by LC-QTOF MS

Dried *P. forrestii* (1.0 g) was powdered and soaked in 70% methanol solution (v/v) for 1 h and extracted under ultrasonic conditions for 40 min at room temperature. The extraction was centrifuged at 3,000 rpm for 15 min and the supernatant was collected after filtering through 0.22 μm membrane for HPLC-QTOF-MS analysis. Sample separation was carried on an Agilent 1260 HPLC system coupled with XBridge BEH C18 Column (5 µm, 4.6 mm × 250 mm) with a mobile phase consisting of methanol containing 0.1% formic acid (A) and water 0.1% formic acid (B). Sample separation was achieved as gradient elution of 5%–100% (A, 0–60 min). Agilent 6530 QTOF MS was used to achieve the identification, positive and negative mode was used in the analysis and the injection volume was 10 μL at the flow rate of 0.3 mL/min (25°C).

### 2.3 Preparation of *Periploca forrestii*

4.0 kg of PF was soaked in water of 10 times the volume for 1 h. After refluxing twice (1.5 h for each time), the extraction was combined and concentrated under vacuum to obtain the extract of *P. forrestii* (PF) for *in vivo* test. The extract was stored in a refrigerator at 4°C and was diluted with water before use.

### 2.4 *In vivo* evaluation

Male Sprague-Dawley (SD) rats (200 ± 20 g) were purchased from Liaoning Changsheng Biotechnology Co., Ltd. All experiments were conducted complying with the Guide of Care and Use of Laboratory Animals approved by Institutional Animal Ethics Committee in China Medical University (permission no. 200403011). On day 0, 0.1 mL aliquots of emulsifying agent CII (2 mg/mL) along with the same volume of CFA (1 mg/mL) were injected intradermally at the base of the tail and left sole, respectively, as the first immunization. On day 7, the second immunization was given. Rats of the normal group were injected with NaCl (0.9%). From day 3 onwards, all rats except the MTX group were orally given for 23 days, and three dosages of PF were 100 mg/kg (L), 200 mg/kg (M), and 400 mg/kg (H), respectively. The MTX group was orally administered one time every 2 days. The arthritis index was scored according to a previous report (Guma et al., 2009).

The index for indicated organs (%) = organ weight (g)/body weight (g)*100%.

### 2.5 Immunohistochemical assessment

Joint tissues were embedded in paraffin wax. Antibodies against rat JAK2 and p-NF-κB were used to analyze the levels of JAK2 and p-NF-κB expression in the ankle joint. The brown color represents the positive result.

### 2.6 Statistical analysis

Data are presented as the means ± SEM. The difference was analyzed using Student’s t-test and ANOVA, and *p* < 0.05 was considered statistically significant.

## 3 Results

### 3.1 Chemical profile of *Periploca forrestii* (PF)

**FIGURE 1:**
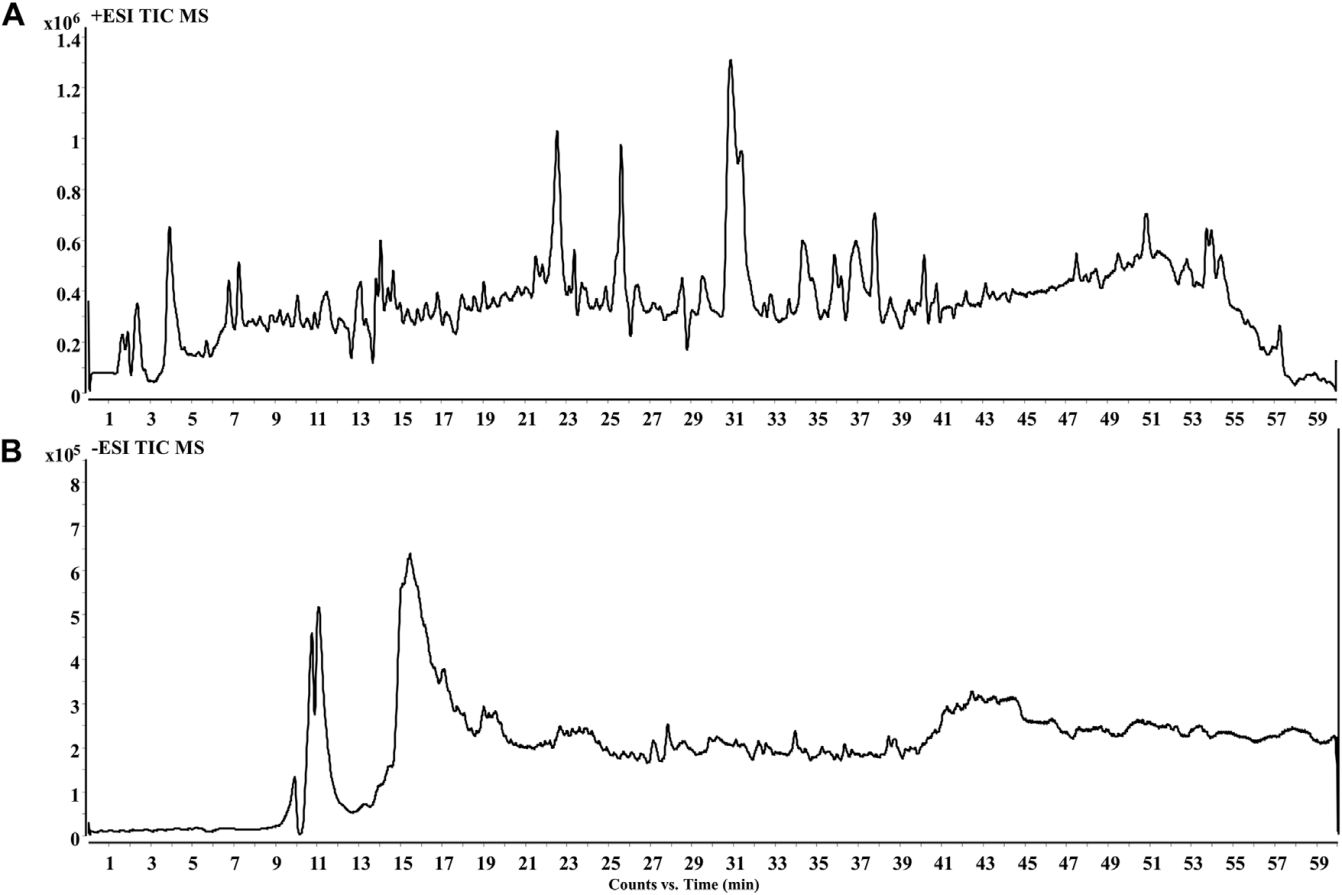
Total-ion chromatogram of PF extracts in positive ion mode **(A)** and negative ion mode **(B)**.

The PF was analyzed by HPLC QTOF-MS for its components to afford basic peak chromatogram (Figure 1). A total of 42 chemical constituents were identified, with terpenoid and flavonoid being the major components (Supplementary Table S1).

### 3.2 Effect of PF on organ indexes

The immune system is a complex network of cells, tissues, and organs that work together to defend the body against harmful pathogens. Two of the most vital organs in the immune system are the spleen and thymus. To determine the effect of PF on the immunological function of these organs, the organs’ relative weights are measured. Rats were sacrificed after the CIA model assessment, and their spleen, thymus, liver, and kidney were carefully weighed to calculate the organ indexes of each rat. The organ indexes provide an indication of the overall health of the immune system in the rats.

**FIGURE 2:**
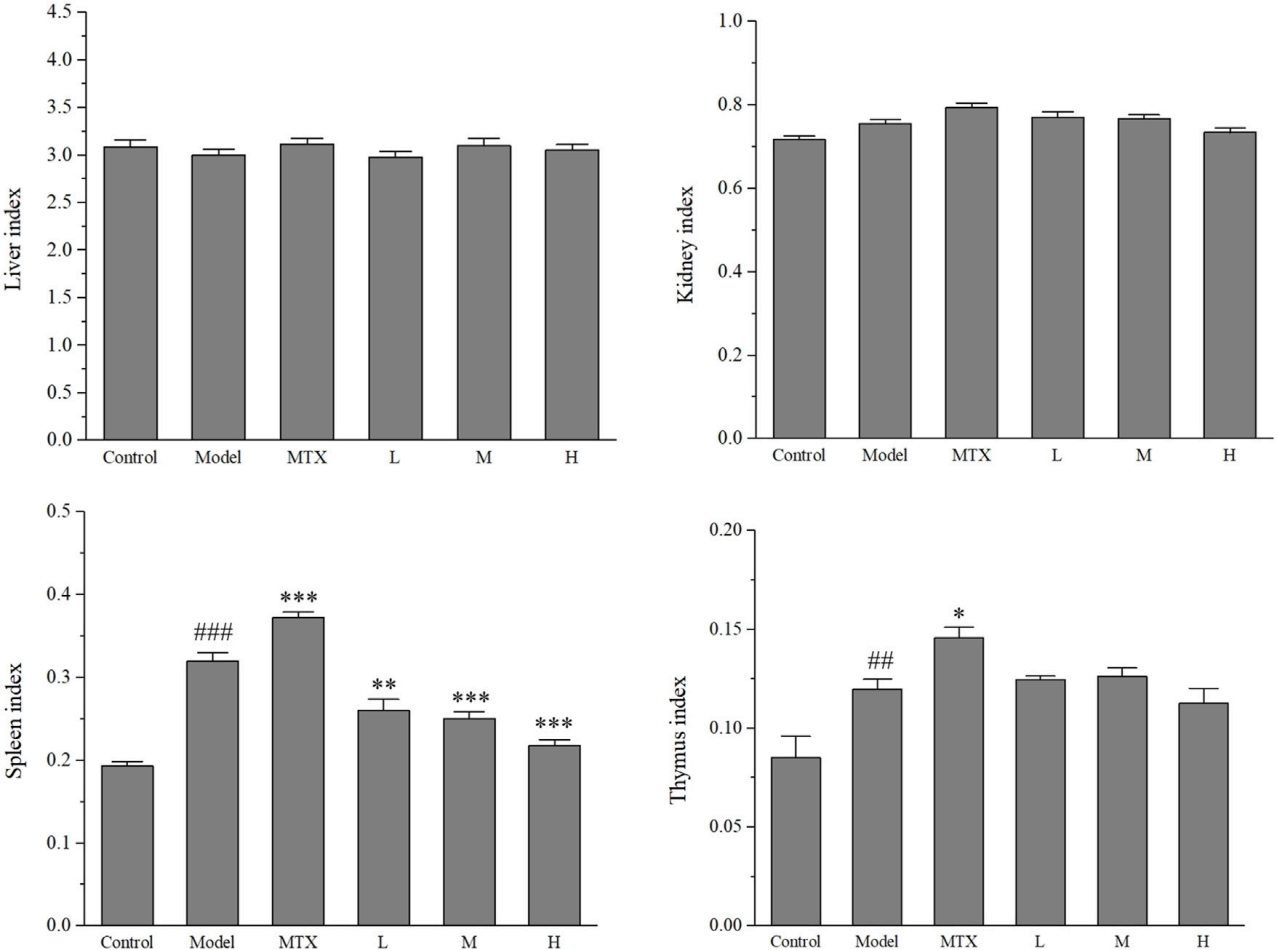
The index of indicated organs of CIA rats. MTX: methotrexate (1.0 mg/kg); L (100 mg/kg), M (200 mg/kg), H (400 mg/kg). Values are means ± the SEM (n = 8). ^##^ *p* < 0.01 and ^###^ *p* < 0.001 vs. control group; ^*^ *p* < 0.05, ^**^ *p* < 0.01 and ^***^ *p* < 0.001 vs. the model group.

The results showed that an evident disparity was observed between control and model (CIA) groups for spleen and thymus indexes. MTX slightly elevated the value of both spleen and thymus indexes, which might be the result of its side effects. Interestingly, PF administration could lower the spleen index compared with both control and MTX groups but failed to restore the thymus index compared with the model group. No alteration was observed for liver and kidney indexes (Figure 2).

### 3.3 PF elicits protective effects on CIA rats

**FIGURE 3:**
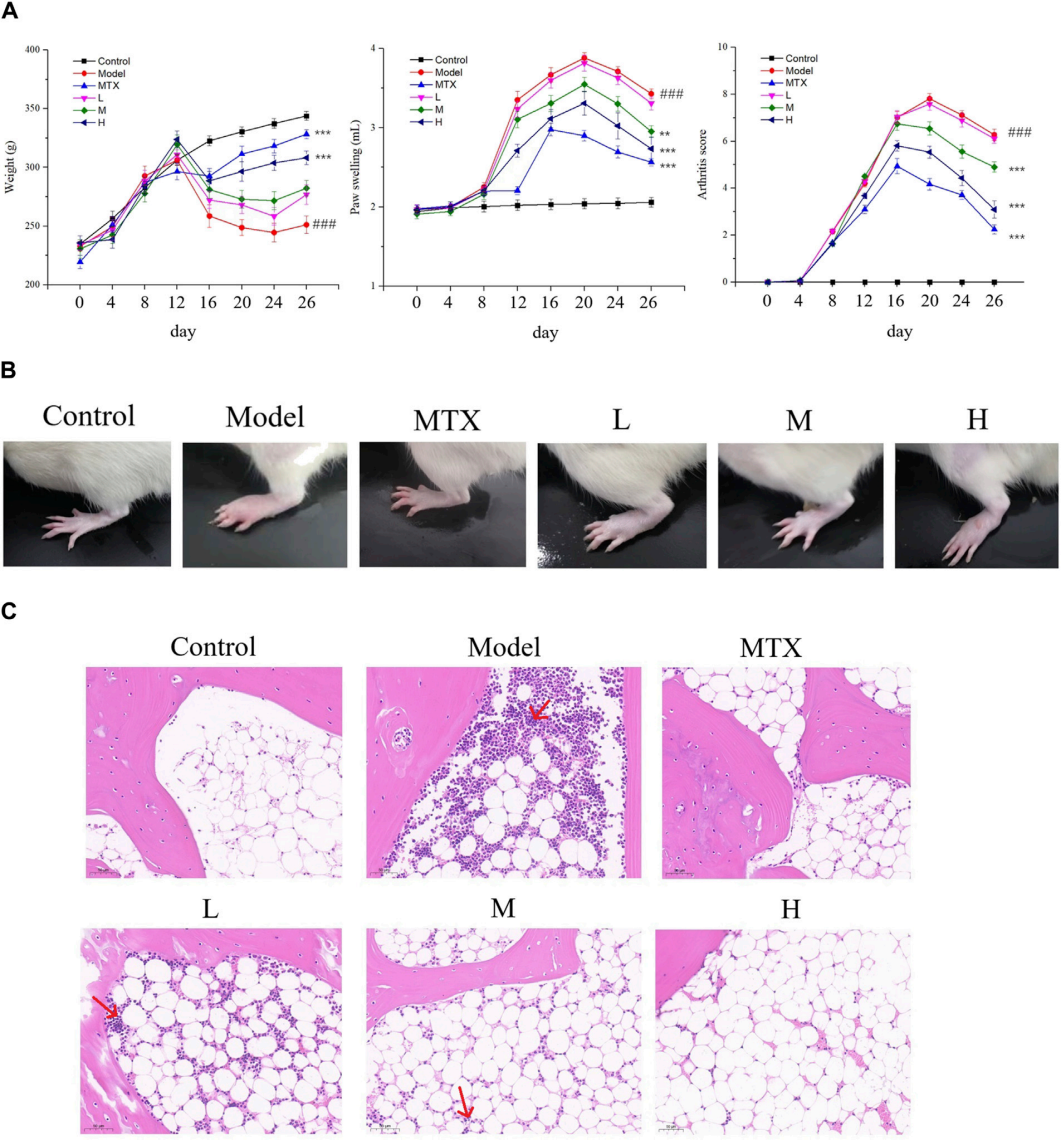
The effect of PF on CIA rats. **(A)** Results of the weight of the body, paw swelling, and the score of arthritis. **(B)** Representative pictures of paws of indicated groups. **(C)** H/E analysis of sections of knee joints in rats (✕200) in indicated groups. ^###^ *p* < 0.001 vs. control group; ^**^ *p* < 0.01 and ^***^ *p* < 0.001 vs. the model group.

To further understand the effect of PF on RA, the Body weight, paw swelling, and arthritis scores of each group were assessed. As a result, Both the positive drug and PF could restore the decreased body weight, ameliorate the paw swelling, and lower the arthritis score for the CIA mice (Figure 3A). Macroscopic assessment indicated an inflamed paw with significant swelling in the model group, while the extent of inflammation and swelling was attenuated by both MTX and PF (especially the high-dosage group) administration (Figure 3B). H/E staining assessment demonstrated a similar result, in which pathological changes of synovial cells and infiltration of inflammatory cells were observed in the model group while these pathological changes were partially reversed by the MTX and PF treatment (Figure 3C), suggesting that PF possesses a protective effect on CIA rats.

**FIGURE 4:**
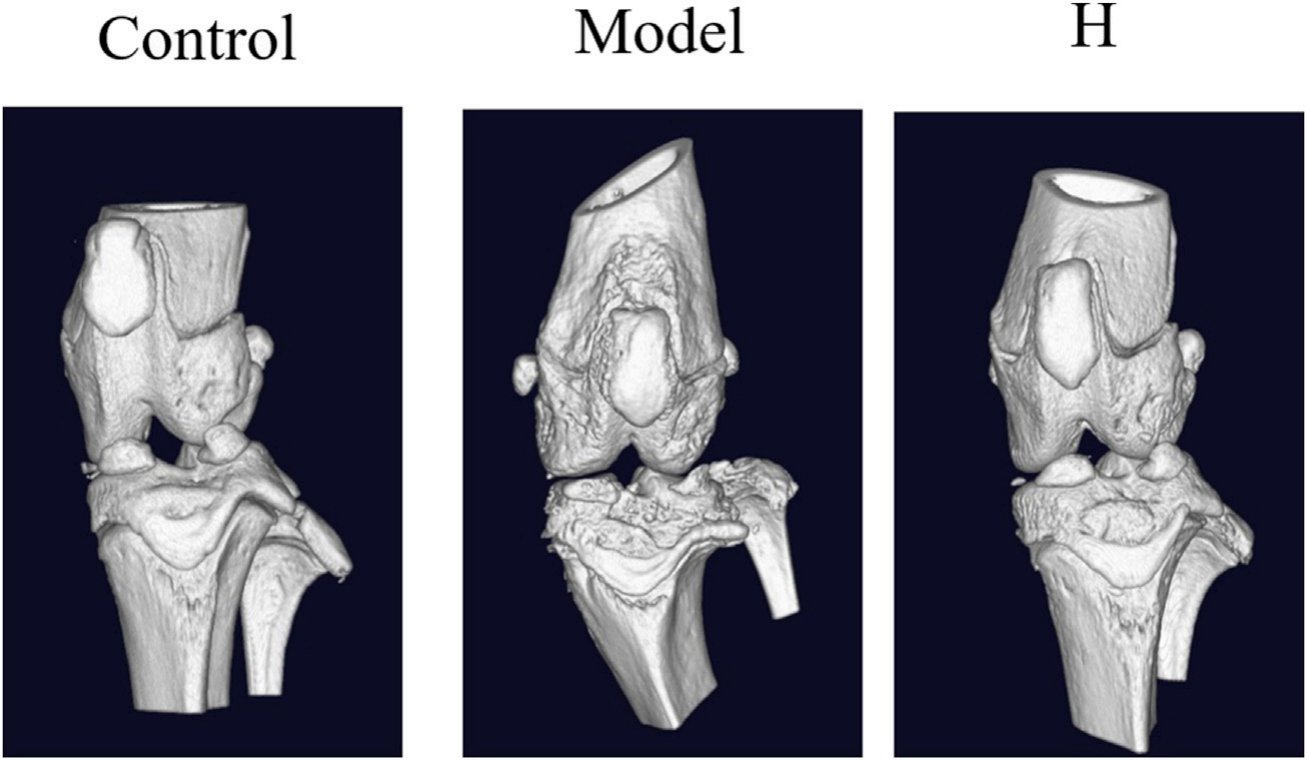
The micro-CT results for sections of CIA rat joints.

To further confirm the protective effect of PF, the micro-CT examination was conducted. The 3D computer-reconstructed results based on the micro-CT results (see Supplementary Data S1) showed severe injuries and scars caused by inflammation in sections of joints for the model group, and in the high-dosage group (400 mg/kg) the inflammation-caused injuries and scars were dramatically ameliorated (Figure 4). All these results substantiated the protective effect of PF on CIA rats and provided a scientific basement for the clinical usage of PF regarding RA.

### 3.4 PF restores serum cytokines of CIA rats

**FIGURE 5:**
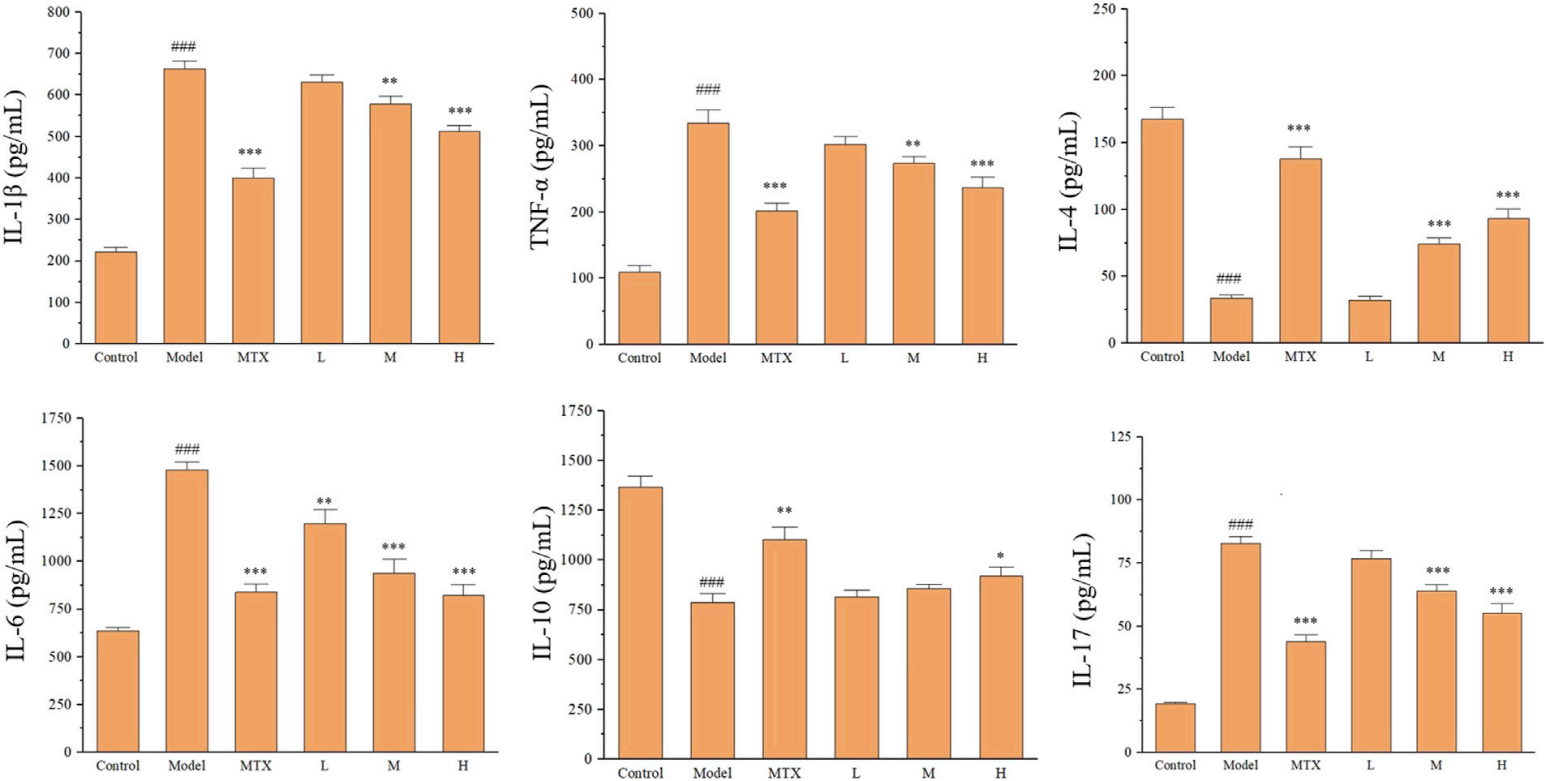
The effect of PF on immune- and inflammation-related cytokines in the serum of CIA rats. ^###^ *p* < 0.001 vs. control group; ^*^ *p* < 0.05, ^**^ *p* < 0.01 and ^***^ *p* < 0.001 vs. the model group.

To further explore the effect of PF on CIA rats, the immune- and inflammation-related cytokines in the serum of CIA rats were tested, including TNF-α, IL-1β, IL-4, IL-6, IL-10, and IL-17 using the ELISA method. The upregulated levels for IL-1β, TNF-α, IL-6, and IL-17 and downregulated levels for IL-4 and IL-10 were detected in the model group. Both MTX and PF administration could restore the ectopic profile of these immune- and inflammation-related serum cytokines (Figure 5).

### 3.5 PF restores Nf-κB phosphorylation of CIA rats

**FIGURE 6:**
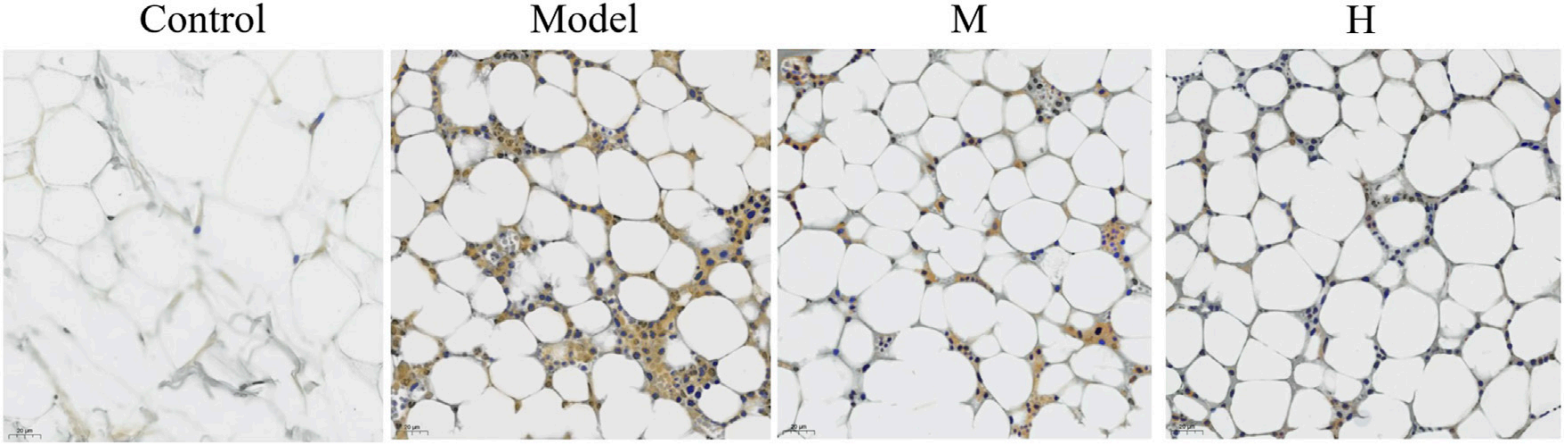
The effect of PF on the phosphorylation of Nf-κB (✕400).

Nf-κB pathway is involved in the pathogenesis of several diseases. Studies have shown that Nf-κB pathway participates in the therapeutic effect of a few traditional medicines (Li et al., 2022). We thus examined the expression of p-Nf-κB using immunohistochemical method. The results showed that the phosphorylation of p-Nf-κB was promoted in the model group while PF treatment partially restored the phosphorylation level of Nf-κB (Figure 6).

### 3.6 PF restores JAK2 pathway of CIA rats

**FIGURE 7:**
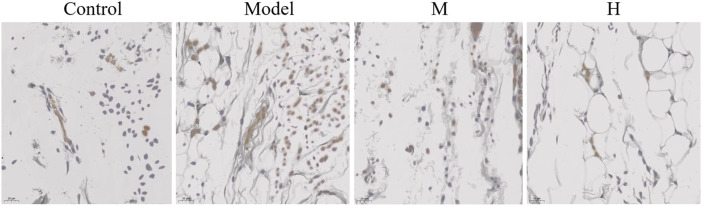
The effect of PF on gene expression of JAK2 (✕400).

In addition to Nf-κB pathway, we further tested the alteration in JAK2 pathway in CIA rats. The results showed that JAK2 expression was elevated in the model group (Figure 7). Similar to what was observed in the p-Nf-κB, the ectopic expression of JAK2 was also partially rescued by PF treatment (Figure 7).

## 4 Discussion

*P. forrestii* is clinically used to treat arthritis, either used alone or in a compound prescription. However, as a plant-derived traditional medicine, its chemical component is, to a large extent, dominantly affected by the location and time of harvest, which determine the quality and thereby the bioactivity of *P. forrestii*. The whole plant of *P. forrestii* is used as a traditional medicine. Unfortunately, the therapeutic effect of the whole plant of *P. forrestii* is so far absent, with only several reports of its stem extract or its specific components like saponins, which do not globally verify the effect and scientific basis for *P. forrestii* regarding its therapeutic effect on RA. On the other hand, several types of components have been identified from *P. forrestii*, including steroids, flavonoids, etc (Sun et al., 2017; Chen et al., 2018). The exact function and mechanism of these components in the *P. forrestii*-elicited therapeutic effect remain elusive. Therefore, more solid investigation should be performed to solve these questions. In this paper, we first verify the function of the whole plant along with its chemical components in the treatment of CIA rats. The results showed that terpenoids and flavonoids are the major components of *P. forrestii*. Following this line of evidence, more studies on terpenoids and flavonoids should be conducted in future studies.

According to previous studies, six signal pathways are involved in the pathogenesis of RA, including MAPK, NF-κB, PI3K, etc. In this paper, we in the first place found the alteration in the serum levels of IL-1β, IL-17, TNF-α, etc. These cytokines can be modulated by MAPKs (McGeachy et al., 2019). Subsequently, we confirmed the ectopic profile of these cytokines could be partially restored by PF treatment, suggesting that the MAPK pathway was involved in the therapeutic effect of PA.

Studies have demonstrated that the expression of NF-κB is considerably higher in the synovial tissue of individuals with rheumatoid arthritis (RA). When NF-κB is highly activated, it can stimulate the production of multiple proinflammatory cytokines, including TNF-α, IL-1β, and IL-6. This, in turn, accelerates the progression of RA. Additionally, the upregulation of pro-inflammatory cytokines can positively regulate the activation of NF-κB, thereby reinforcing a vicious cycle that further aggravates RA progression (Noort et al., 2015). In this paper, we obtained a similar result that the abnormal phosphorylation in the CIA rats could be rescued by PF treatment.

RA is a chronic autoimmune disorder that affects joints, resulting in synovitis, which is characterized by inflammation of the synovial membrane. The synovial membrane in RA patients exhibits a range of inflammatory responses, including the activation of cytokines and adhesion molecules. These responses are related to specific transcription factors and signal transduction pathways. Research has shown that pro-inflammatory cytokines phosphorylate JAK, leading to the activation of the STAT protein. Persistent inflammation and the severity of joint destruction in RA patients are linked to the transcription of STAT genes (Rosillo et al., 2016). In this paper, we found that JAK2 was the potential target of PF. Interestingly, JAK inhibitors are a recent discovery in the field of rheumatoid arthritis treatment. These targeted small molecules are designed to block the intracellular signaling that causes inflammation and joint damage. For example, tofacitinib is a particularly promising drug. It is a medication that specifically inhibits the JAK pathway, which is a key player in the inflammatory response. Developed in 2012, tofacitinib has been granted marketing approval by the US Food and Drug Administration. These results suggest that it is of great potential to explore novel JAK inhibitors from PF.

## Data Availability

The datasets presented in this study can be found in online repositories. The names of the repository/repositories and accession number(s) can be found in the article/Supplementary Material.
