# Supplementary material for: Therapeutic effect of Periploca forrestii on collagen-induced arthritis in rats through JAK2/Nf-κB pathway
Source: Front Pharmacol. 2024 May 22;15:1415392. doi: 10.3389/fphar.2024.1415392 (PMC11150650; doi:10.3389/fphar.2024.1415392)
Supplement: Supplementary file 1 [file Table1.DOCX]

Supplementary Material

*Periploca forrestii* Schltr alleviates arthritis in rats through JAK2/Nf-κB pathway

Zhenyi Zhang^1^, Yingchun Li^1^, Jian Wu^2^, Jihong Zhang^1^, Ning Chen^2^, Ning Zhang^1*^

^1^ Department of Rheumatology and Immunology, Shengjing hospital of China Medical University, China

^2^ College of Pharmacology, Harbin University of Commerce, China

*** Correspondence:**Zhang Ning
cmuzn@sohu.com

**Table S1** Chemical constituents idenfied by HPLC-QTOF MS.

| Putative Identification | Formula | Base Peak | Mass | Ion Mode | RT (min) |
| --- | --- | --- | --- | --- | --- |
| Neoeriocitrin | C_27_H_32_O_15_ | 579.1719 | 596.1741 | [M+H]^+^ | 9.644 |
| Geniposide | C_17_H_24_O_10_ | 741.2601 | 388.1370 | [M+H]^+^ | 9.93 |
| Sesamin | C_20_H_18_O_6_ | 707.2117 | 354.1103 | [M-H]^-^ | 10.871 |
| Steviol Hydroxydehydrostevic acid | C_20_H_30_O_3_ | 323.1972 | 318.2195 | [M+H]^+^ | 11.579 |
| IristectorinA | C_23_H_24_O_12_ | 493.1336 | 492.1268 | [M+H]^+^ | 11.882 |
| Citropten | C_11_H_10_O_4_ | 207.0649 | 206.0579 | [M+H]^+^ | 12.050 |
| Tiliroside | C_30_H_26_O_13_ | 577.1350 | 594.1373 | [M+H]^+^ | 12.706 |
| Gastrodin | C_13_H_18_O_7_ | 805.2939 | 286.1052 | [M+H]^+^ | 12.824 |
| Xanthatin | C_15_H_18_O_3_ | 515.2409 | 246.1256 | [M+H]^+^ | 12.942 |
| Isofraxidin | C_11_H_10_O_5_ | 223.0605 | 222.0528 | [M+H]^+^ | 13.06 |
| 8-Dihydroxyanthraquinone | C_14_H_8_O_4_ | 223.0380 | 240.0423 | [M+H]^+^ | 14.238 |
| Dihydroartemisinin | C_15_H_24_O_5_ | 267.1591 | 284.1624 | [M+H]^+^ | 14.759 |
| 5-Feruloylquinic acid | C_17_H_20_O_9_ | 369.1192 | 368.1107 | [M+H]^+^ | 15.230 |
| (+)-Nortrachelogenin | C_20_H_22_O_7_ | 357.1338 | 374.1366 | [M+H]^+^ | 15.348 |
| Hesperetin | C_16_H_14_O_6_ | 285.0758 | 302.0790 | [M+H]^+^ | 15.584 |
| 8-Heptamethoxyflavone” | C_22_H_24_O_9_ | 415.1400 | 432.1420 | [M+H]^+^ | 16.004 |
| 2''-O-Rhamnosylicariside II | C_33_H_40_O_14_ | 643.2393 | 660.2418 | [M+H]^+^ | 16.055 |
| Cimifugin | C_16_H_18_O_6_ | 613.2295 | 306.1103 | [M+H]^+^ | 16.358 |
| Gramine | C_11_H_14_N_2_ | 371.2221 | 174.1157 | [M+H]^+^ | 16.644 |
| Methyl 4-Methoxycinnamate | C_11_H_12_O_3_ | 193.0856 | 192.0786 | [M+H]^+^ | 16.997 |
| Periplogenin | C_23_H_34_O_5_ | 391.2484 | 390.2406 | [M+H]^+^ | 17.653 |
| Armepavine | C_19_H_23_NO_3_ | 314.1758 | 313.1678 | [M+H]^+^ | 18.124 |
| Asebotin | C_22_H_26_O_10_ | 433.1501 | 450.1526 | [M+H]^+^ | 18.292 |
| Irisflorentin | C_20_H_18_O_8_ | 387.1089 | 386.1002 | [M+H]^+^ | 18.360 |
| Andropanolide | C_20_H_30_O_5_ | 351.2172 | 350.2093 | [M+H]^+^ | 18.528 |
| Engeletin | C_21_H_22_O_10_ | 417.1191 | 434.1213 | [M+H]^+^ | 18.646 |
| Auraptene | C_19_H_22_O_3_ | 299.1645 | 298.1569 | [M+H]^+^ | 18.999 |
| Caffeic acid | C_9_H_8_O_4_ | 163.0385 | 180.0423 | [M+H]^+^ | 21.473 |
| Coniferol | C_10_H_12_O_3_ | 181.0855 | 180.0786 | [M+H]^+^ | 23.122 |
| Periplocymarin | C_30_H_46_O_8_ | 535.3286 | 534.3193 | [M+H]^+^ | 23.778 |
| Corticosterone | C_21_H_30_O_4_ | 329.2122 | 346.2144 | [M+H]^+^ | 24.485 |
| Rhapontigenin 3'-O-glucoside | C_21_H_24_O_9_ | 403.1399 | 420.1420 | [M+H]^+^ | 27.715 |
| Dihydrocapsaicin | C_18_H_29_NO_3_ | 308.2224 | 307.2147 | [M+H]^+^ | 29.230 |
| Polydatin | C_20_H_22_O_8_ | 373.1294 | 390.1315 | [M+H]^+^ | 30.424 |
| Schisandrin | C_24_H_32_O_7_ | 415.2136 | 432.2148 | [M+H]^+^ | 31.552 |
| Protopine | C_20_H_19_NO_5_ | 336.1248 | 353.1263 | [M+H]^+^ | 32.157 |
| 14-Deoxyandrographolide | C_20_H_30_O_4_ | 335.2207 | 334.2144 | [M+H]^+^ | 33.016 |
| Alisol A | C_30_H_50_O_5_ | 473.3648 | 490.3658 | [M+H]^+^ | 34.193 |
| 10-Gingerol | C_21_H_34_O_4_ | 351.2546 | 350.2457 | [M+H]^+^ | 35.607 |
| Pseudolaric acid B | C_23_H_28_O_8_ | 415.1772 | 432.1784 | [M+H]^+^ | 36.263 |
| Higenamine | C_16_H_17_NO_3_ | 507.2295 | 271.1208 | [M+H]^+^ | 36.785 |
| (-)-Deoxypodophyllotoxin | C_22_H_22_O_7_ | 399.1456 | 398.1366 | [M+H]^+^ | 39.326 |


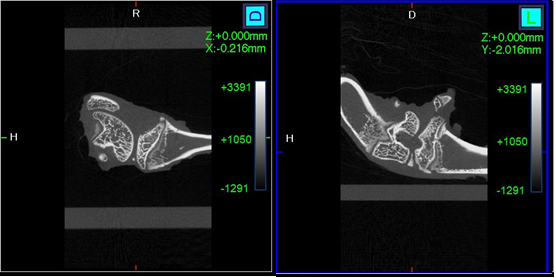


1. Micro-CT for the model group


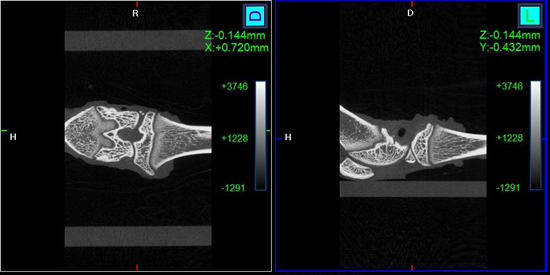


(B) Micro-CT for the control group


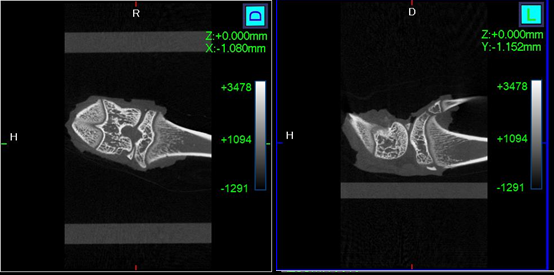


(C) Micro-CT for the H group

**Figure S1.** Micro-CT results. A, model group; B, control group; C, H group.
